# Endophytic *Pseudomonas* sp. from *Agave palmeri* Participate in the Rhizophagy Cycle and Act as Biostimulants in Crop Plants

**DOI:** 10.3390/biology11121790

**Published:** 2022-12-09

**Authors:** Qiuwei Zhang, Kathryn L. Kingsley, James F. White

**Affiliations:** Department of Plant Biology, School of Environmental and Biological Sciences, Rutgers University, New Brunswick, NJ 08901, USA

**Keywords:** plant growth-promoting bacteria, plant–microbe interactions, endophytes, biostimulants, rhizophagy cycle, confocal microscopy

## Abstract

**Simple Summary:**

As a result of increasing public pushback against chemical fertilizers in agriculture, farmers and agricultural companies are becoming more interested in environmentally friendly alternatives for boosting crop yields. These alternatives, called biostimulants, come from a variety of sources, one of which is the plant itself. Endophytes, defined as microorganisms that live within plant tissues without causing any apparent harm, have emerged as potential substitutes for traditional fertilizers. In this study, we showed that a group of bacterial endophytes taken from Palmer’s agave exhibit growth-promoting effects in a variety of crops. We used confocal microscopy to track one of these endophytes from Palmer’s agave and demonstrated that it enters other plants’ tissues and participates in the rhizophagy cycle, a process by which microbes cycle between the soil and roots and bring nutrients into the plant. Tracking of this endophyte also suggests a mechanism by which endophytes or their cell contents may be moved throughout the plant. These results provide further insights into the mechanisms behind the way in which endophytes promote growth in plants.

**Abstract:**

Plant growth-promoting bacteria are generating increasing interest in the agricultural industry as a promising alternative to traditional chemical fertilizers; however, much of the focus has been on rhizosphere bacteria. Bacterial endophytes are another promising source of plant growth-promoting bacteria, and though many plants have already been prospected for beneficial microbes, desert plants have been underrepresented in such studies. In this study, we show the growth-promoting potential of five strains of endophytic *Pseudomonas* sp. isolated from *Agave palmeri*, an agave from the Sonoran Desert. When inoculated onto Kentucky bluegrass, clover, carrot, coriander, and wheat, endophytic *Pseudomonas* sp. increased seedling root lengths in all hosts and seedling shoot lengths in Kentucky bluegrass, carrot, and wheat. Transformation of the *Pseudomonas* sp. strain P3AW to express the fluorescent protein mCherry revealed that *Pseudomonas* sp. becomes endophytic in non-native hosts and participates in parts of the rhizophagy cycle, a process by which endophytic bacteria cycle between the soil and roots, bringing in nutrients from the soil which are then extracted through reactive oxygen-mediated bacterial degradation in the roots. Tracking of the *Pseudomonas* sp. strain P3AW also provided evidence for a system of endophyte, or endophyte cell content, transport via the vascular bundle. These results provide further evidence of the rhizophagy cycle in plants and how it relates to growth promotion in plants by biostimulant bacteria.

## 1. Introduction

As the world moves further into the 21st century, the agricultural industry must grapple with the twin challenges of increasing crop yield to feed a growing global population while simultaneously reducing large-scale human and ecosystem damage from overreliance on agrochemicals and irrigation. As such, there has been increasing interest from agricultural companies in developing and utilizing biostimulants as an alternative to traditional chemical fertilizers to help enhance crop productivity without incurring additional damage to the environment [1,2,3,4].

While there are many types of biostimulants, bacterial biostimulants have garnered much attention due to their natural occurrence in soils and within plant tissues. There have been many studies focused on using plant growth-promoting rhizobacteria (PGPR) such as *Rhizobium* spp., *Bacillus* spp., *Klebsiella* spp., and *Arthrobacter* spp. as biostimulants [5,6,7,8], but another promising source of bacterial biostimulants are endophytes, which are defined as microbes that live within plant tissues without causing apparent harm to their hosts [9,10]. Endophytes have been shown to increase host growth, though the mechanisms behind this growth-promoting ability are still not entirely understood [11,12,13,14]. Though endophytes from a variety of hosts, such as tomato (*Solanum lycopersicum*), rice (*Oryza sativa* L.), wheat (*Triticum* sp.), eagle fern (*Pteridium aquilinum*), Indian olive (*Olea ferruginea* Royle), and creeping woodsorrel (*Oxalis corniculate*) have been investigated for their growth-promoting properties [12,15,16,17,18], much less is known about the growth-promoting properties of desert plant endophytes.

As farmlands continue to experience more extreme weather events, particularly drought, as a result of climate change [19,20,21,22,23], crop yields are predicted to decline [24,25]. Decreasing yields and increasing human populations, especially in arid regions, necessitates the discovery and development of new technologies that can alleviate the impacts of extreme and prolonged heat waves on crops while simultaneously boosting productivity [26,27]. Endophytes from desert plants may be one such technology that can be employed against drought-induced crop damage. Desert plants, such as *Agave palmeri,* are unique in that they are specialized to survive in drought-like conditions with high temperatures, high salt concentrations, and low rainfall. While they have a variety of physical adaptations that aid in their survival in arid locales, the endophytes that they have cultivated might be a part of their survival strategy as well. Endophytes have been shown to increase host tolerance to a variety of abiotic stresses and can confer such resistance even to non-native host species [28,29,30]. 

A previous meta-analysis of desert plant endophytes has shown that the Proteobacteria are the largest phylum of bacterial endophytes in desert plants, with *Pseudomonas* being the most abundant genus within the Proteobacteria endophytes found in desert plants [31]. Fluorescent *Pseudomonas* spp. in particular are well-known as biocontrol agents and plant growth promoters [32,33,34,35,36], which makes them ideal biostimulant candidates. In addition, they are commonly found in both soils and plant tissues [37,38,39]. Their ability to proliferate in both environments may be a sign that members of this genera contribute to plant growth promotion via the “rhizophagy cycle”.

First coined by Paungfoo-Lohienne et al. (2013) [40], the rhizophagy cycle hypothesis posits that important plant nutrients may be acquired in the roots via a process by which endophytic bacteria cycle between the soil and roots, bringing in nutrients from the soil which are then extracted through reactive oxygen-mediated degradation of the microbes within the root cells [40,41,42]. Endophytes participating in the rhizophagy cycle may be responsible for bringing in important plant nutrients from the rhizosphere. It is currently unknown if all bacteria have the capacity to participate in the rhizophagy cycle or if participation is limited only to certain genera or phyla.

In this study, we sought to describe the plant growth-promoting properties of several strains of fluorescent and non-fluorescent *Pseudomonas* sp. isolated from *A. palmeri* growing in the Sonoran Desert and demonstrate their participation in the rhizophagy cycle.

## 2. Materials and Methods

### 2.1. Collection, Isolation, and Characterization of Endophytes from A. palmeri Seeds

Seed pods were randomly selected and collected from wild *A. palmeri* in Arizona, USA at AZ State Rt. 289, Mile 3 Marker (31.424525, −111.010064) and stored at 4 °C. To isolate endophytes from agave seeds, the seeds were surface-sterilized with 4% NaOCl for 1 h under continuous agitation. After 1 h, the NaOCl was decanted and excess NaOCl was removed from seed surfaces by rinsing with sterile DI H_2_O for 5 min; this was repeated 6 times or until the scent of chlorine had disappeared. Surface-sterilized seeds were placed onto Yeast Extract Sucrose (YES) agar (10 g yeast extract, 10 g sucrose, and 15 g agar in 1 L) and allowed to germinate. Bacterial growth was subcultured onto Luria-Bertani (LB) agar (Sigma-Aldrich, St. Louis, MO, USA).

After isolation, bacterial strains were tested for 6 characteristics: fluorescence, lipopeptide production, casein digestion, gelatinase activity, nitrogen fixation, and phosphate solubilization activity.

Fluorescence was determined by growing the strains at room temperature on LB agar. After 48 h, the plates were placed under a UV lamp and visually assessed for signs of fluorescence.

Lipopeptide production was determined by inoculating the strains into YES broth (10 g yeast extract, and 10 g sucrose in 1 L) at room temperature. After 24 h, a 500 μL sample of inoculated broth was placed onto Parafilm M and visually assessed for changes in droplet shape as a result of surfactant-related surface tension compared to the uninoculated broths. 

Casein digestion activity was determined by subculturing the strains on Skim Milk Agar (14 g dry milk powder, 5 g pancreatic digest of casein, 2.5 g yeast extract, 1 g sucrose, and 15 g agar in 1 L) and allowing them to grow at room temperature. After 48 h, the plates were evaluated for any changes in transparency around the bacterial streak.

Gelatinase activity was determined by inoculating the strains into test tubes filled with Nutrient Gelatin Agar (120 g gelatin, 5 g peptone, and 3 g beef extract in 1 L) via an agar stab and allowing them to grow at 37 °C for 48 h. The test tubes were then placed in an ice bath for 30 min and evaluated for gelatin liquefaction. 

Nitrogen fixation was determined by subculturing the strains on Jensen’s N-Free Medium (20 g sucrose, 2 g CaCO_3_, 1 g K_2_HPO_4_, 0.5 g MgSO_4_, 0.5 g NaCl, 0.1 g FeSO_4_, 0.005 g Na_2_MoO_4_, and 15 g agar in 1 L) at room temperature. After 1 week, the plates were evaluated for any signs of bacterial growth. 

Phosphate solubilization was determined by subculturing the strains on Pikovskaya’s Agar (10 g dextrose, 0.5 g yeast extract, 5 g Ca_3_(PO_4_)_2_, 0.5 g K_2_HPO_4_, 0.2 g KCl, 0.1 g MgSO_4_, 0.0001 g MnSO_4_, 0.0001 g FeSO_4_, and 15 g agar in 1 L) at room temperature. After 1 week, the plates were evaluated for any changes in transparency around the bacterial streak.

### 2.2. Identification of Endophytic Bacteria from A. palmeri

Purified bacterial cultures were grown and maintained on LB agar at room temperature. At a timepoint 24 h prior to DNA collection, the endophytes were subcultured onto fresh LB agar and allowed to grow at room temperature. DNA was collected from endophyte cultures using the GenElute Bacterial Genomic DNA Kit (Sigma-Aldrich). A total of 4 genes were chosen for sequencing: 16S rRNA, atpD (ATP synthase β-subunit), carA (carbamoyl phosphate synthase small subunit), and recA (recombinase A). The genes were amplified via PCR using the primers listed in Table 1 and sent to Genewiz (South Plainfield, NJ, USA) for sequencing. Sequences were compared to the GenBank database using NCBI nBLAST and phylogenetic tree construction in order to obtain an identification.

Maximum likelihood (ML) phylogenetic trees were constructed using the MEGA11: Molecular Evolutionary Genetics Analysis version 11 software [44]. ML analyses were performed using the Kimura-2 parameter substitution model with discrete gamma distribution and invariant sites for the 16S rRNA gene, and the Tamura 3-parameter substitution model with discrete gamma distribution for the atpD, carA, and recA genes. The ML Heuristic model used was Nearest-Neighbor-Interchange. Clade stability was assessed using a bootstrap analysis with 1000 replicates. 

### 2.3. Surface Sterilization and Inoculation of Crop Seeds

Five crop species were used to test for biostimulant properties: Kentucky bluegrass ‘Midnight’ (*Poa pratensis*), red clover (*Trifolium pratense*), cilantro (*Coriandrum sativum*), carrot (*Daucus carota* var *sativa*), and winter wheat (*Triticum* sp.). Red clover was used to evaluate rhizophagy participation. 

Clover and coriander seeds were surface-sterilized with 4% NaOCl for 30 min or 1 h, respectively, under continuous agitation. After the pre-requisite amount of time had passed, the sodium hypochlorite was decanted and excess NaOCl was removed from seed surfaces by rinsing with sterile DI H_2_O for 5 min; this was repeated 6 times or until the scent of chlorine had disappeared. 

Kentucky bluegrass, carrot, and wheat seeds were first placed in an oven at 50 °C for 48 h before being surface-sterilized as described above for the coriander seeds.

### 2.4. Biostimulation Testing

Crop seeds were inoculated either with individual strains of *Pseudomonas* sp. or inoculated with a *Pseudomonas* sp. mix composed of strains P3AW, AY2, WCY, and WC. To inoculate the seeds, the bacterial strains were grown on LB agar 24–48 h prior to inoculation. A bacterial suspension of the desired strain(s) was created by suspending the agar-grown strains in sterile DI water until they reached a concentration of 10^8^ CFU/mL. The seeds were then soaked in the suspension for 10 min and placed into the growth substrate. All plants were grown at room temperature under a 12 h light/dark cycle in the laboratory under normal laboratory conditions.

The growth substrate was composed of Sunshine Mix #8 (Sun Gro Horticulture, Agawam, MA, USA) that had been filtered through a 2 mm sieve. A total of 100 mL of filtered soil mix was added to a Magenta box and sterilized by autoclaving for 60 min a total of three times, with 24 h elapsing between each autoclave session. A total of 31 mL of sterile DI H_2_O was added to each box to moisten the soil. A total of 10 seeds were placed into each box. Containers were separated into two groups: uninoculated and inoculated, with each treatment having at least 3 replicates.

The average seed germination per treatment was calculated by counting the number of seeds that had germinated per box.

All statistical analyses were performed using R Statistical Software (v.4.2.2) and R Commander [45,46,47,48].

### 2.5. Drought Resistance Testing

Wheat seeds were inoculated with a *Pseudomonas* sp. mix composed of strains P3AW, AY2, WCY, and WC. Inoculation of the seeds followed the same protocol as described in the biostimulation tests. The growth substrate used was the same as described in the biostimulation tests. Containers were separated into 4 treatment groups: irrigated with uninoculated seeds, drought with uninoculated seeds, irrigated with *Pseudomonas* inoculation, and drought with *Pseudomonas* inoculation, each with 3 replicates. Both groups were irrigated with 10 mL of water, with the irrigated group watered every 3 days and the drought group watered every 6 days.

The average seed germination per treatment was calculated by counting the number of seeds that had germinated per box.

All statistical analyses were performed using R Statistical Software and R commander (v.4.2.2) [45,46,47,48].

### 2.6. Transformation of Pseudomonas sp. for mCherry Expression

To induce mCherry expression in *Pseudomonas* sp. isolated from *A. palmeri*, the isolates were transformed with plasmid pSEVA237R_Pem7, a self-replicating, broad-host-range plasmid that is not inserted chromosomally and does not contain any transposons [49]. pSEVA237R_Pem7 contains an mCherry gene expressed under the constitutive promoter Pem7 and a kanamycin resistance gene, allowing for the continuous production of mCherry under kanamycin selection. The plasmid was extracted from *Escherichia coli* DH5α using the NucleoSpin Plasmid Mini Kit (Macherey-Nagel, Düren, Germany).

*Pseudomonas* sp. cells were grown overnight in 5–10 mL of a 1:1 Potato Dextrose Broth (PDB) + Nutrient Broth (NB) mixture (PDB: Sigma-Aldrich; NB: Sigma-Aldrich) at 23 °C (OD_600_ = 2.5) and collected via centrifugation at 14,000 rpm for 5 min. The cells were washed thrice with 1 mL of 300 mM sucrose, then resuspended in 100 μL of 300 mM sucrose. A total of 500 ng of plasmid DNA was added to 100 μL of cell suspension and the mixture was transferred to a 2 mm electroporation cuvette (Bio-Rad, Hercules, CA, USA). The cells were electroporated at 2.5 kV/cm, 25 μF, and 200 Ω using a Gene Pulser (Bio-Rad, Hercules, CA, USA), then immediately resuspended in 1 mL of PDB + NB and incubated at 23 °C for 2 h. After incubation, 100 μL of cell suspension was plated onto 1:1 Nutrient Agar (NA) + Potato Dextrose Agar (PDA) plates (NA: 8 g NB, and 15 g agar in 1 L; PDA: Sigma-Aldrich) amended with 50 μg/mL of kanamycin. Visibly pink colonies were cultured further on PDA + NA agar plates amended with kanamycin, and mCherry production was confirmed using a confocal microscope (Zeiss LSM 710; Carl Zeiss, Oberkochen, Germany).

### 2.7. Confocal Microscopy of Intracellular Bacteria

Clover seeds were inoculated with *Pseudomonas* sp. P3AW:*mCherry* (henceforth referred to as P3AW:*mCherry*) using the same procedure as described in the inoculation section. Inoculated seeds were placed into agarose plates amended with 75 μg/mL kanamycin to induce the continued production of mCherry. The seeds were allowed to grow in the agar for 3–4 days in a 12 h day–night cycle before they were subjected to high levels of CO_2_ (5–6 g of CO_2_ per 1 L of air) for 24 h.

Leaf tissue samples were lightly scraped and stained with Calcofluor White M2R (Sigma-Aldrich, St. Louis, MO, USA) and SYTO13 (Life Technologies, Carlsbad, CA, USA) before being viewed on a Zeiss LSM 710 confocal microscope. Root tissue samples were stained with Calcofluor White M2R and SYTO13 but not scraped prior to viewing. The 405 nm, 488 nm, and 594 nm lasers were used to excite the Calcofluor White M2R, SYTO13, and mCherry stains, respectively. Double staining with SYTO13 and mCherry was utilized to distinguish between mCherry-producing P3AW:*mCherry* and other background artifacts that also fluoresce red, such as auto-fluorescing chloroplasts.

## 3. Results

### 3.1. Categorization and Identification of Endophytic Bacteria

Eleven strains of bacteria were isolated from the seeds of *Agave palmeri* and then tested for their growth-promoting capabilities. Five strains in particular showed greater promise as biostimulants, with the following biochemical properties listed in Table 2. Strains P3AW, P3BW, AY2, and WCY are yellow-green in color, while strain WC is a transparent, almost-white color.

**Figure 1:**
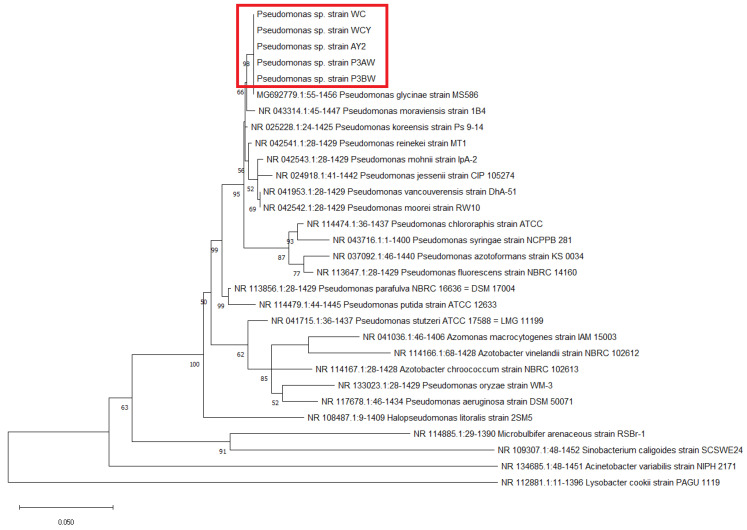
Maximum likelihood phylogenetic tree of *Pseudomonas* sp. strains based on the 16S rRNA gene. The number at the branches indicates the percentage of occurrences of that branch over 1000 bootstrap replications, with values under 50% hidden. *Pseudomonas* sp. strains from *A. palmeri* are circled by the red box.

**Figure A1:**
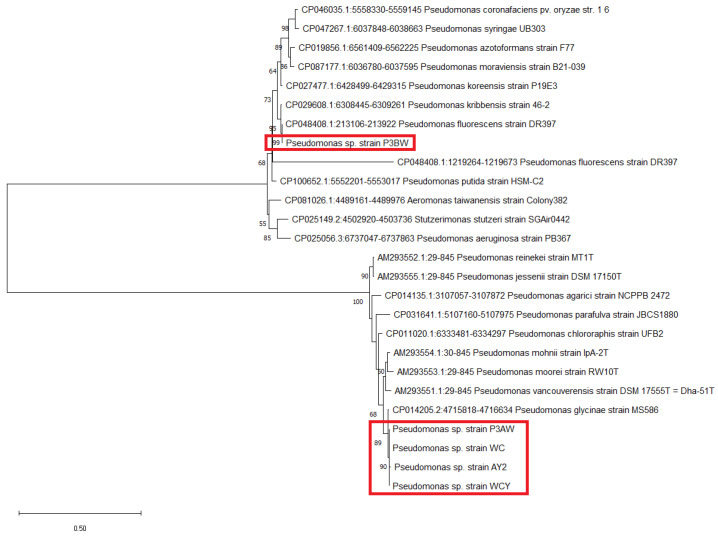
Maximum likelihood phylogenetic tree of *Pseudomonas* sp. strains based on the atpD gene. The number at the branches indicates the percentage of occurrences of that branch over 1000 bootstrap replications, with values under 50% hidden. *Pseudomonas* sp. strains from *Agave palmeri* are circled by the red box.

**Figure A2:**
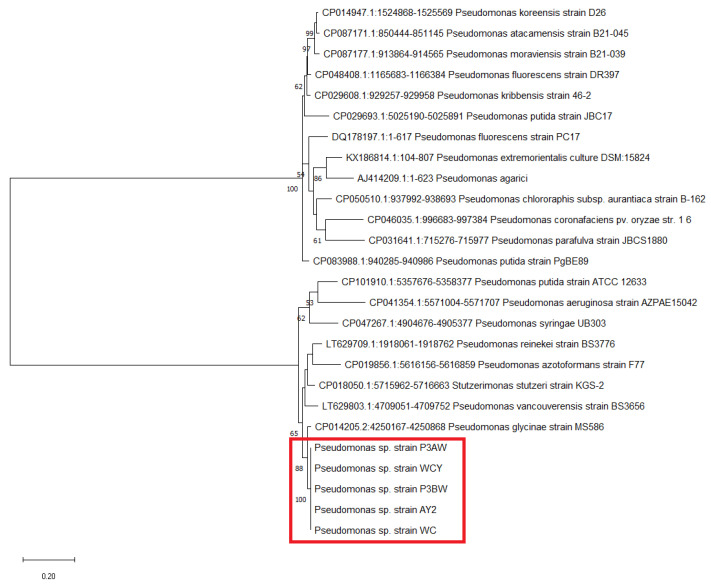
Maximum likelihood phylogenetic tree of *Pseudomonas* sp. strains based on the carA gene. The number at the branches indicates the percentage of occurrences of that branch over 1000 bootstrap replications, with values under 50% hidden. *Pseudomonas* sp. Strains from *Agave palmeri* are circled by the red box.

**Figure A3:**
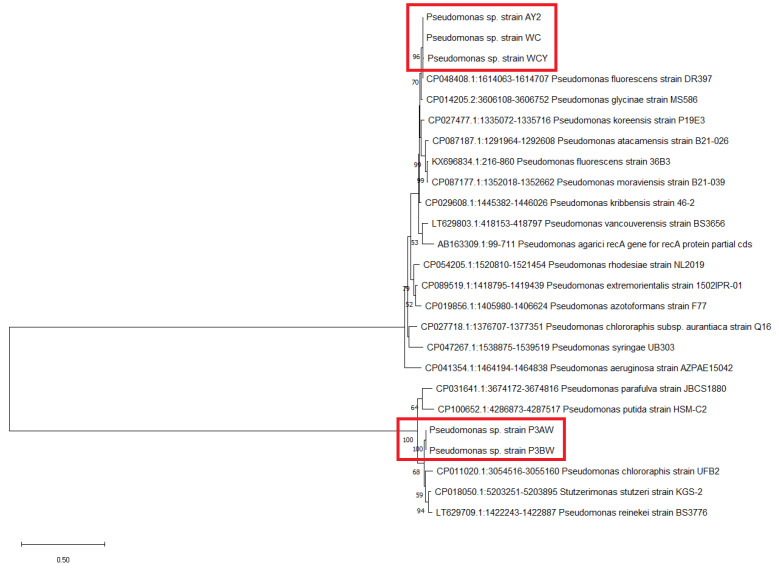
Maximum likelihood phylogenetic tree of *Pseudomonas* sp. Strains based on the recA gene. The number at the branches indicates the percentage of occurrences of that branch over 1000 bootstrap replications, with values under 50% hidden. *Pseudomonas* sp. strains from *Agave palmeri* are circled by the red box.

Molecular-based identification of the five strains were conducted using the 16S rRNA, atpD, carA, and recA genes. All strains were identified as part of the genus *Pseudomonas* based on GenBank database matches, however, a species-level identification could not be achieved due to close clustering of the five strains with other *Pseudomonas* species (Figure 1, Figure A1, Figure A2 and Figure A3). The closest match to the five strains was *Pseudomonas glycinae*, isolated from soybean rhizospheres. The 16S rRNA sequences were uploaded to GenBank under the accession numbers listed in Table 2.

### 3.2. Inoculation of Seeds with Individual Pseudomonas sp.

Individual *Pseudomonas* sp. were inoculated onto Kentucky bluegrass and red clover seeds to determine the efficacy of individual strains as biostimulants and to ascertain if there were any negative effects of applying individual strains to crops. After 8 weeks (Kentucky bluegrass) or 6 weeks (red clover) of growth, seedlings roots and shoots were measured to evaluate growth-promoting capabilities.

**Figure 2:**
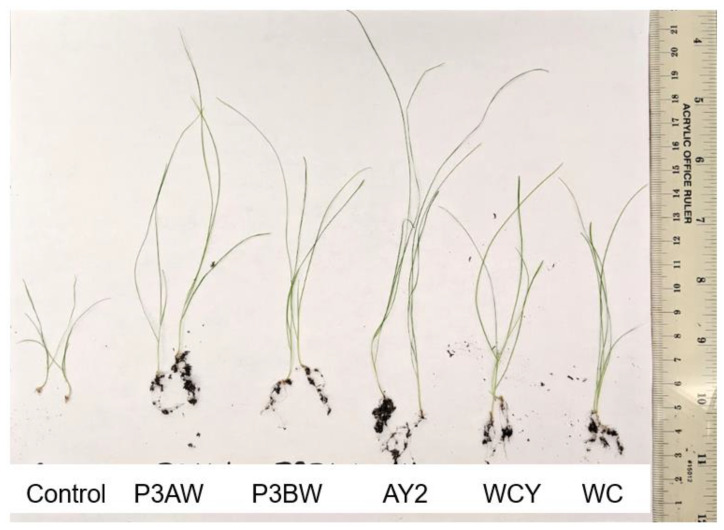
Visual comparison of Kentucky bluegrass seedlings inoculated with individual *Pseudomonas* sp. strains. Total number of seedlings germinated = 25, 25, 22, 23, 20, and 23, from left to right.

A visual comparison of the Kentucky bluegrass seedlings (Figure 2) revealed that uninoculated control seedlings had little to no root mass, while inoculated seedlings had substantially longer roots with greater soil adsorption. In addition, inoculated seedlings had much longer blades compared to the control seedlings. Overall, strains P3AW, P3BW, WCY, and WC appeared to be comparable in terms of growth-promotional effects, while strain AY2 appeared to confer greater growth-promoting effects.

**Figure 3:**
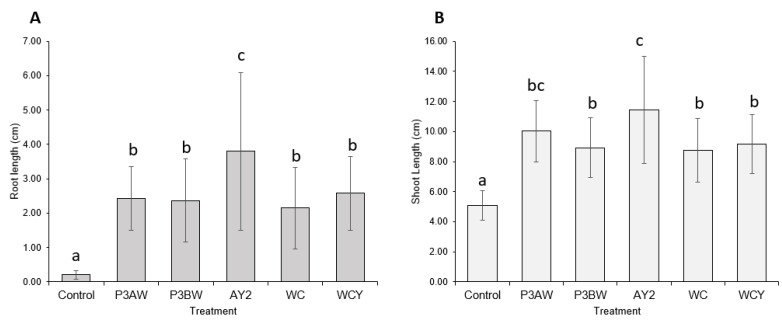
Kentucky bluegrass growth promotion by individual *Pseudomonas* sp. strains. (**A**) Root and (**B**) shoot lengths of 8-week-old Kentucky bluegrass seedlings inoculated with different *Pseudomonas* sp. strains. Error bars represent standard deviation. Different letters represent significant differences among treatments according to Tukey’s HSD test (*p* ≤ 0.05).

Statistical analysis supported the notion that the addition of *Pseudomonas* sp. strains to Kentucky bluegrass enhances seedling growth, as significant increases in both the root length (Figure 3A) and shoot length (Figure 3B) of inoculated seedlings were seen. As with the visual comparison, strains P3BW, WCY, and WC appeared to be comparable in terms of growth-promoting effects. Strain AY2 appeared to be significantly better than all other strains for root-length promotion but only significantly better than strains P3BW, WC, and WCY for shoot-length promotion. No notable reductions in the germination rate between the control and inoculated seedlings was observed, though strain AY2 seemed to boost the average germination rate by 16% compared to non-inoculated plants (Table A1).

**Figure 4:**
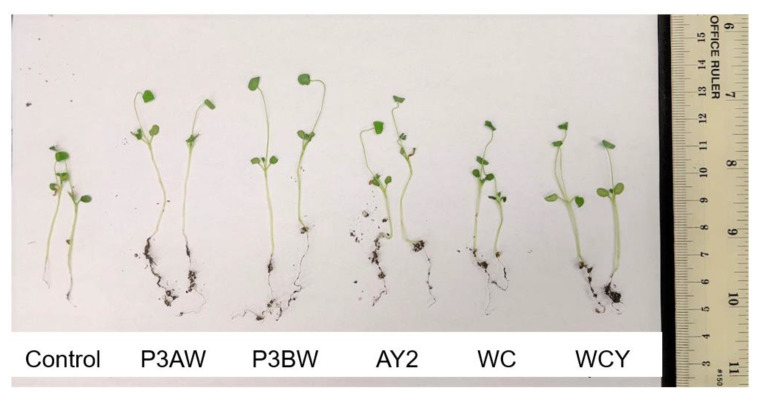
Visual comparison of red clover seedlings inoculated with individual *Pseudomonas* sp. Strains. Total number of seeds germinated = 14, 18, 21, 22, and 19, from left to right.

A visual comparison of red clover seedlings revealed similar results to the Kentucky bluegrass seedlings (Figure 4). Again, the inoculated seedlings had longer roots and greater soil adsorption compared to the uninoculated control seedlings. Strains AY2, WC, and WCY appeared to confer comparable root-length enhancements, while strains P3AW and P3BW appeared to confer the greatest root-length enhancements. However, it was impossible to visually determine if there were any noticeable enhancements to shoot length.

**Figure 5:**
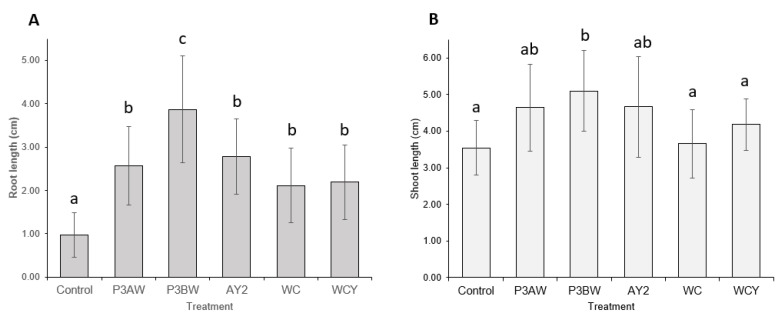
Red clover growth promotion by individual *Pseudomonas* sp. Strains. (**A**) Root and (**B**) shoot lengths of 6-week-old red clover seedlings inoculated with different *Pseudomonas* sp. Strains. Error bars represent standard deviation. Different letters represent significant differences among treatments according to Tukey’s HSD test (*p* ≤ 0.05).

Statistical analysis confirmed that inoculation with any of the five *Pseudomonas* sp. strains significantly improved the root length of red clover seedlings, though strain P3BW conferred the greatest root-length enhancements (Figure 5A). However, only strain P3BW significantly improved shoot length (Figure 5B). No notable reductions in the germination rate between the control and inoculated seedlings was observed, though strains P3BW, AY2, and WCY appeared to increase the average germination rate by 60%, 47%, and 33%, respectively (Table A1).

### 3.3. Inoculation of Seeds with a Mixture of Pseudomonas sp.

Since the endophytic *Pseudomonas* sp. were isolated from the same *A. palmeri* seeds, we wanted to test if the strains could be used together as an endophytic mix or synthetic community instead of as individual endophytes. Since the efficacy of the strains as a mixture of bacteria was unknown, we did not wish to overload the seedlings by inoculating them with too many strains. Strain P3BW was chosen to be omitted due to its similarity to strain P3AW in terms of chemical activity, as we wanted to test non-redundant strains to assess their plant growth-promoting effects as a microbial community.

Due to seed availability and difficulties in fully sterilizing seeds, preliminary studies on the effects of single inoculation on other seedlings were conducted on agarose plates to determine which other hosts would be best. Carrot and coriander were chosen due to an increased gravitropic response in inoculated seedlings, and, as such, carrot and coriander seeds were inoculated with a mix of strains P3AW, AY2, WC, and WCY. After 2 weeks of growth, seedling roots and shoots were measured.

**Figure 6:**
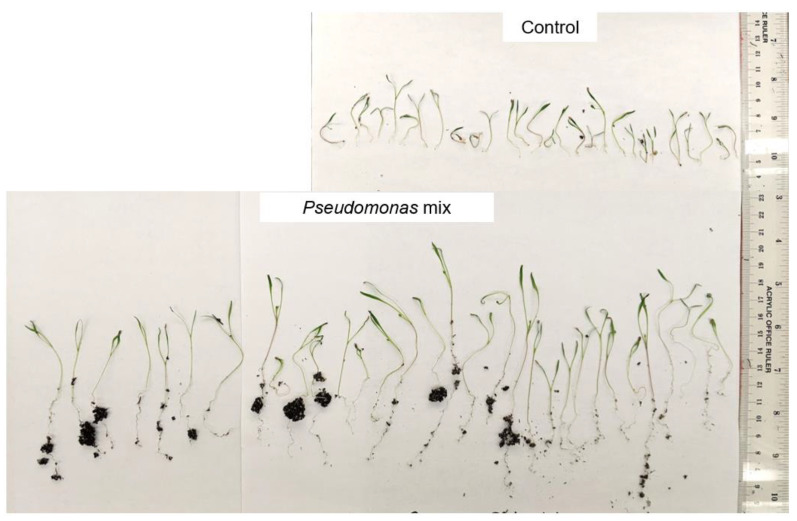
Visual comparison of carrot seedlings inoculated with a *Pseudomonas* sp. mix. Total number of seedlings germinated = 29, and 31, from top to bottom.

**Figure 7:**
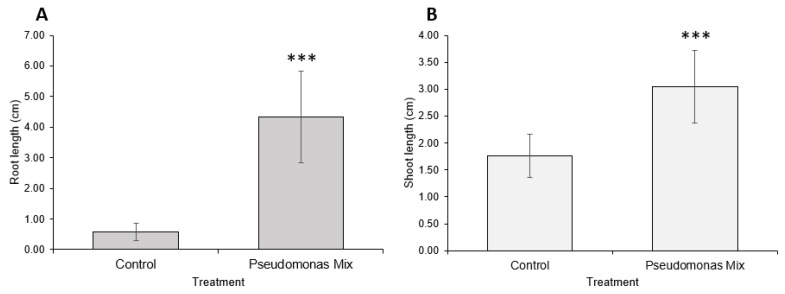
Carrot growth promotion by a mix of *Pseudomonas* sp. strains. (**A**) Root and (**B**) shoot lengths of 2-week-old carrot seedlings inoculated with *Pseudomonas* sp. P3AW, AY2, WC, and WCY. Error bars represent standard deviation. Asterisks represent the *p* value of the Student’s T-test conducted between the control and the experimental group. ns = *p* > 0.05, * = *p* ≤ 0.05, ** = *p* ≤ 0.01, *** = *p* ≤ 0.001.

A visual comparison of inoculated and uninoculated carrot seedlings (Figure 6) revealed that inoculated seedlings had noticeably greater root and shoot lengths, greater soil adsorption, and larger cotyledons. Some inoculated seedlings had already grown the first true leaf, while none of the uninoculated seedlings exhibited true leaf growth. Statistical analysis confirmed that inoculation with a mix of *Pseudomonas* sp. Strains significantly improved the root (Figure 7A) and shoot lengths (Figure 7B) of carrot seedlings. However, the improvement in root length was superior to the improvement in shoot length. In addition, inoculation with a *Pseudomonas* sp. mix was able to increase the germination rate by 25% (Table A1).

**Figure 8:**
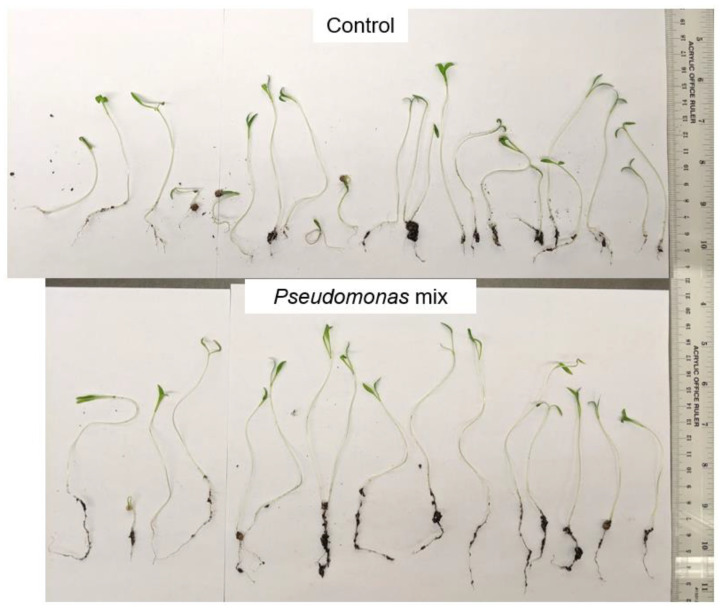
Visual comparison of coriander seedlings inoculated with a *Pseudomonas* sp. mix. Total number of seedlings germinated = 22, and 16, from top to bottom.

A visual comparison of inoculated and uninoculated coriander seedlings (Figure 8) reveals that inoculated seedlings had noticeably greater root length, greater soil adsorption, and larger cotyledons. The germination rate was slightly reduced by 10% when inoculated with a *Pseudomonas* sp. mix (Table A1). This may be due to the way coriander seeds germinate, where a single seed produces two seedlings. The failure of one seed to germinate would lead to a loss of two seedlings instead of one, doubling any differences in the germination rate.

**Figure 9:**
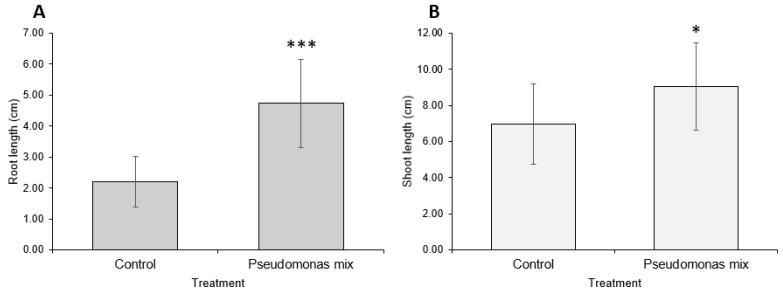
Coriander growth promotion by a mix of *Pseudomonas* sp. strains. (**A**) Root and (**B**) shoot lengths of 2-week-old coriander seedlings inoculated with *Pseudomonas* sp. P3AW, AY2, WC, and WCY. Error bars represent standard deviation. Asterisks represent the *p* value of the Student’s T-test conducted between the control and the experimental group. ns = *p* > 0.05, * = *p* ≤ 0.05, ** = *p* ≤ 0.01, *** = *p* ≤ 0.001.

Statistical analysis confirmed that inoculation with a mix of *Pseudomonas* sp. strains significantly improved root (Figure 9A) and shoot lengths (Figure 9B) of coriander seedlings. However, shoot-growth promotion was weaker compared to root-growth promotion.

### 3.4. Assessment of Drought Resistance Induced by a Mixture of Pseudomonas sp.

Wheat was chosen as the model crop for drought resistance testing due to its status as one of the world’s most popular crops, and due to its susceptibility to drought stress. After 3 weeks of growth, seedling root and shoots were measured.

**Figure 10:**
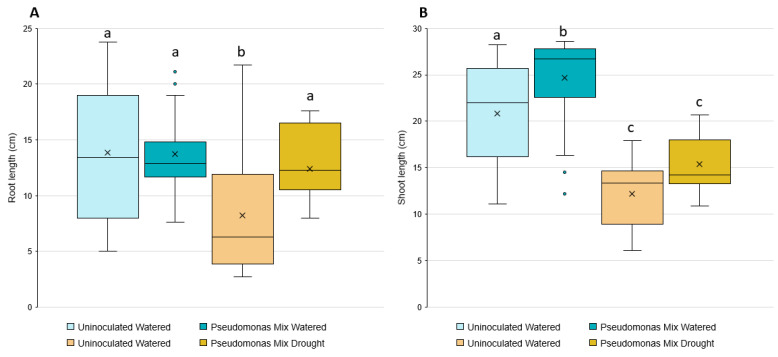
Induction of wheat drought resistance by a mix of *Pseudomonas* sp. strains. Box and whisker plots are used to show the (**A**) root and (**B**) shoot lengths of 3-week-old inoculated and uninoculated wheat seedlings in the presence or absence of drought stress. Blue colored bars represent seedlings grown in irrigated conditions. Yellow bars represent seedlings grown in drought conditions. Boxes encompass the interquartile range (Q_1_–Q_3_), whiskers outline the minimum and maximum data points. Xs inside boxes indicate the mean, dots outside the whiskers indicate outliers. Different letters represent significant differences among treatments according to Tukey’s HSD test (*p* ≤ 0.05).

Statistical analysis showed that inoculated seedlings have significantly longer root lengths when grown in drought conditions (Figure 10A); however, no significant changes in root length were observed in seedlings grown in irrigated conditions. The inoculation-related increase in root length under drought conditions only increased root length relative to the uninoculated seedlings also grown under drought conditions, allowing the inoculated seedlings to reach similar root lengths as the irrigated groups. 

The shoot-growth analysis results (Figure 10B) were the opposite of the root-length results—the inoculated seedlings only showed a significant increase in irrigated conditions, not drought conditions. While there was a small increase in shoot length between inoculated and uninoculated seedlings grown under drought stress, it was not a statistically significant difference.

Germination rates seemed to be greatly affected by the presence of *Pseudomonas* sp. (Table A2). This is especially notable in drought conditions, where inoculated boxes averaged 5.5 germinated seeds per box while uninoculated boxes averaged 8 germinated seeds per box, a decrease of 31%. The average number of dead seedlings per box remained the same regardless of inoculation (Table A2).

### 3.5. Tracking Pseudomonas sp. in Clover Tissues Using mCherry

**Figure 11:**
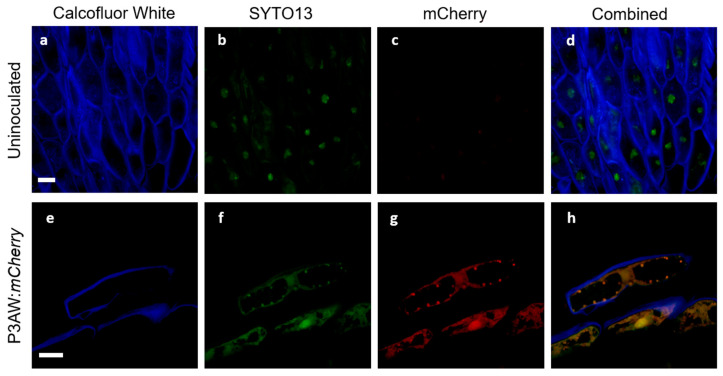
Validation of confocal microscopy as a method to track P3AW:*mCherry* in clover cells. (**a**–**d**) Sloughed-off root cells of uninoculated plants. (**e**–**g**) Sloughed-off root cells of P3AW:mCherry-inoculated plants. (**a**,**e**) Fluorescence of Calcofluor-White-stained cell walls using the 405 nm laser. (**b**,**f**) Fluorescence SYTO-13-stained DNA using the 488 nm laser. (**c**,**g**) Fluorescence of mCherry using the 594 nm laser. (**d**,**h**) Combined fluorescence images using all three lasers. No fluorescence was detected in either (**b**) or (**c**). Scale bar = 10 µm.

To ensure that the triple-staining system involving Calcofluor White, SYTO13, and mCherry would function in clover and allow for the tracking of P3AW:*mCherry* in clover tissues with minimal false positives, a validation study was first conducted in the root tissues. The validation test involved viewing inoculated and uninoculated clover root caps under a confocal microscope to ensure that each stain localized to the correct areas and to determine if there were any auto-fluorescing artifacts within clover roots. The results of the test are shown in Figure 11.

In both uninoculated and inoculated roots, emissions could be detected in the low-wavelength blue (Figure 11a,e) and medium-wavelength green spectrum (Figure 11b,f). The blue emissions can be found surrounding the cells and are in the shape of the cell wall, suggesting that Calcofluor White can be used to stain and view the cell wall in the triple-staining system. The green emissions can be found scattered within the cells, but are particularly strong near the nucleus, suggesting that SYTO13 can be used to non-specifically stain DNA in the triple-staining system.

In uninoculated roots, very faint emissions could be detected in the high-wavelength red spectrum (Figure 11c), while in the inoculated roots, strong emissions could be detected in the red spectrum (Figure 11g). The relatively low level of red spectrum emissions in the uninoculated roots compared to the high level of emissions in the inoculated roots suggests that there is little to no red autofluorescence within clover root tissues. The overlap in green and red emissions between Figure 11b,c suggests that some small amount of red emissions in clover root tissue is due to spectral crosstalk.

The combined channel images of the uninoculated root tissue (Figure 11d) shows no orange or gold areas indicative of true overlap between the green spectrum of SYTO13 and the red spectrum of mCherry, indicating no areas where DNA and mCherry are expressed simultaneously. Meanwhile, the P3AW:*mCherry*-inoculated roots (Figure 11h) shows much overlap between the green and red spectra, indicating that there are several areas where DNA and mCherry overlap. The presence of such an overlap can be contributed to intact P3AW:*mCherry* or the simultaneous existence of free-floating DNA and mCherry. The lack of overlap in uninoculated tissues combined with the presence of an overlap in the inoculated tissues shows that the triple-staining system is valid for the detection of mCherry and P3AW:*mCherry* in clover roots.

Following the successful validation of the triple-staining system, we attempted to discern where exactly P3AW:*mCherry* localizes inside clover roots. To distinguish between free-floating DNA and mCherry and intact P3AW:*mCherry*, we looked for circular areas of green and red spectrum overlap around 1 μm in size, which are present in inoculated tissues (Figure 11h) but not in uninoculated tissues (Figure 11d).

**Figure 12:**
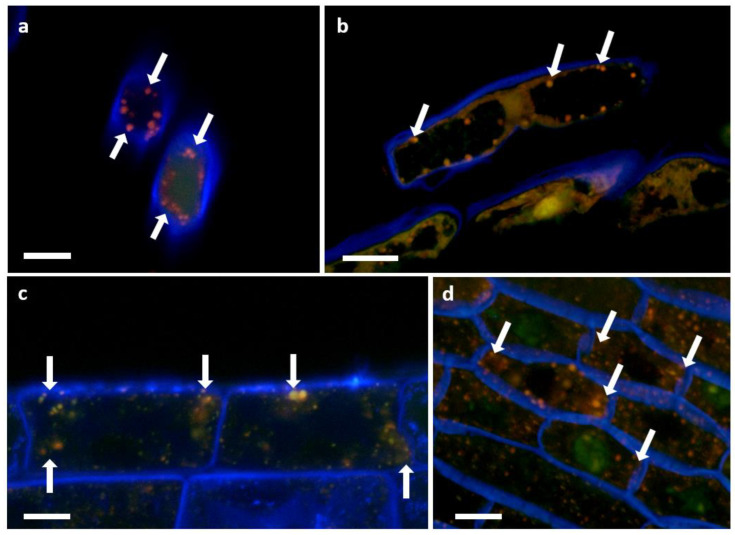
P3AW:*mCherry* localizes to the cell periphery near the cell wall. (**a**) Front view and (**b**) side view of sloughed-off root cells of P3AW:*mCherry*-inoculated clover. Side view of (**c**) epidermal cells and (**d**) sclerenchyma cells of P3AW:*mCherry*-inoculated clover. The arrows point to the spherical L-forms of P3AW:*mCherry*, which can be found near the cell periphery, close to the cell wall. Scale bar = 10 μm.

Images from the confocal microscopy on inoculated root cells are shown in Figure 12. The presence of intact P3AW:*mCherry* inside the borders of the cell walls of sloughed-off root cap cells (Figure 12a,b), epidermal cells (Figure 12c), and sclerenchyma cells (Figure 12d) shows that P3AW:*mCherry* becomes endophytic inside of clover roots and can enter clover root cells. Their exact position within the root cells is harder to distinguish, as the cell membrane was not stained. In root cap and epidermal cells, it appears that P3AW:*mCherry* is found close to the cell wall (Figure 12a–c), while in sclerenchyma cells, it appears that P3AW:*mCherry* is found around the nucleus or the central vacuole (Figure 12d). Three-dimensional confocal videos of root cap cells (Videos S1 and S2) appear to confirm that P3AW:*mCherry* is found in the cell periphery.

**Figure 13:**
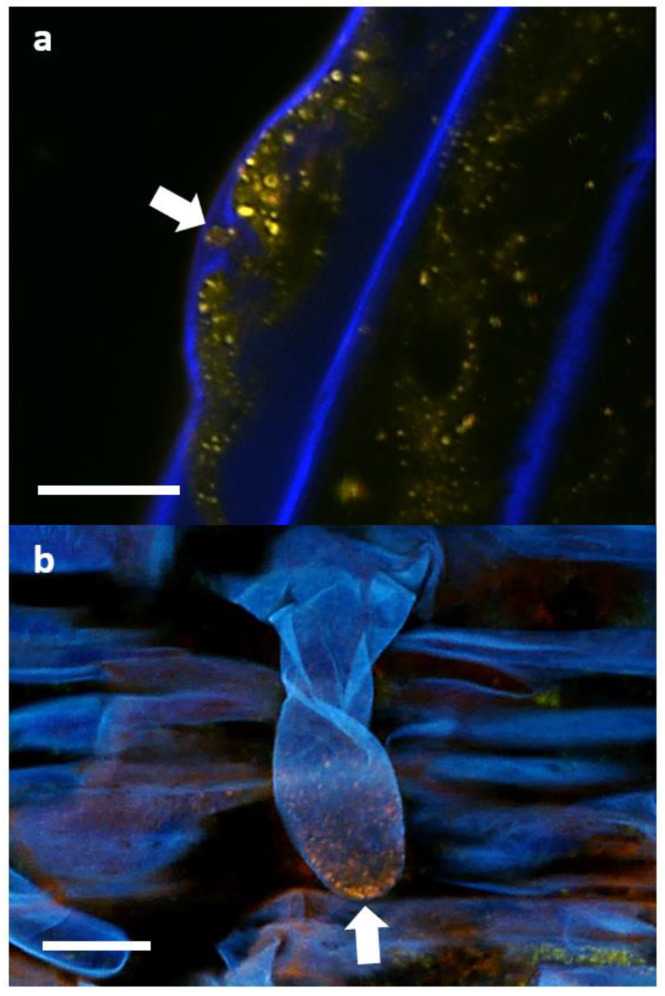
P3AW:*mCherry* gathers at the root hair tips at the root hair tips of inoculated clover roots. P3AW:*mCherry* L-forms can be seen in (**a**) a root hair primordial and (**b**) a fully formed root hair. The arrows point to the root hair tip. Scale bar = 10 μm.

**Figure 14:**
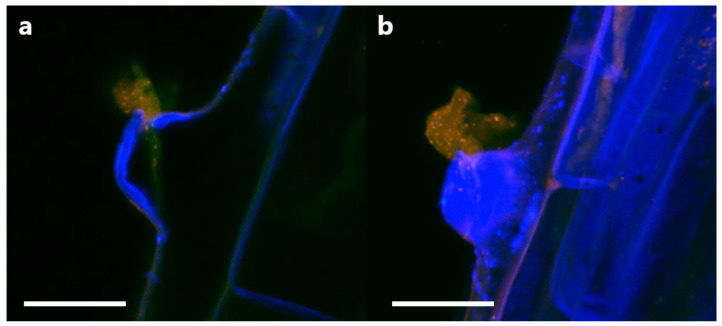
P3AW:mCherry are expelled at the root hair tips. (**a**) A 2D image of intact P3AW:*mCherry* cells, along with free-floating mCherry and DNA, escaping a root hair primordial. (**b**) The same scene is rendered as a 3D confocal stack. Scale bar = 15 μm.

In order to determine if P3AW:*mCherry* participates in the rhizophagy cycle, we also investigated its localization in root hairs specifically. Confocal microscopy revealed that the circular L-forms of P3AW:*mCherry* are found within both the developing (Figure 13a) and developed (Figure 13b) root hairs, and that they appear to congregate at the root hair tips. P3AW:*mCherry* was also observed exiting the root hair through an opening in the cell wall (Figure 14). The bacteria that exit the root hair in this manner are surrounded by a cloud of free-floating mCherry and DNA, which may be a result of bacterial degradation by the plant.

These results suggest that P3AW:*mCherry* does participate in at least part of the rhizophagy cycle, namely the phases where the bacteria become endophytic inside plant cells, are partially degraded by the plant, and then exit the root via the root hairs.

**Figure 15:**
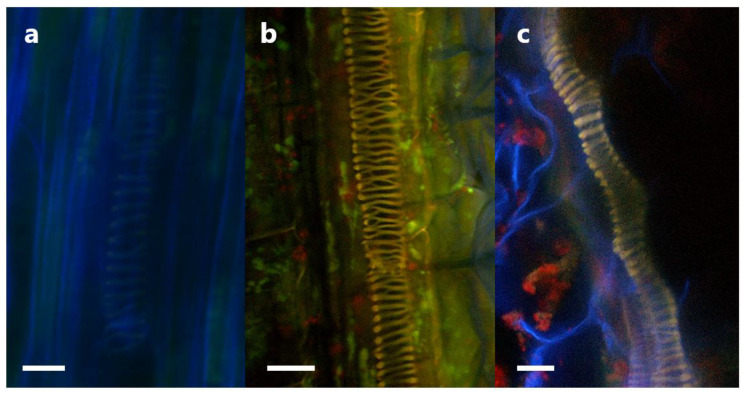
DNA and mCherry in the xylem of P3AW:*mCherry* inoculated clover plants. (**a**) Root xylem of an uninoculated plant. (**b**) DNA and mCherry in the root xylem of an inoculated plant. (**c**) DNA and mCherry in the cotyledon xylem of an inoculated plant. Scale bar = 15 μm.

If P3AW:*mCherry* is indeed degraded for nutrients in the plant roots, then its cell contents, namely mCherry and DNA, should also be trackable through plant tissues as those contents are moved through the plant. Indeed, a mixture of mCherry and DNA was found in the xylem of inoculated roots and shoots (Figure 15). It is unknown whether the mCherry and DNA are free-floating in water or contained as part of intact P3AW:*mCherry* since there appear to be no distinct circular L-forms. This suggests that P3AW:*mCherry*, or its cell contents, are transported from the roots to the shoots via the xylem. Additionally, non-degraded P3AW:*mCherry* was found in the vascular bundle (Figure A1).

**Figure 16:**
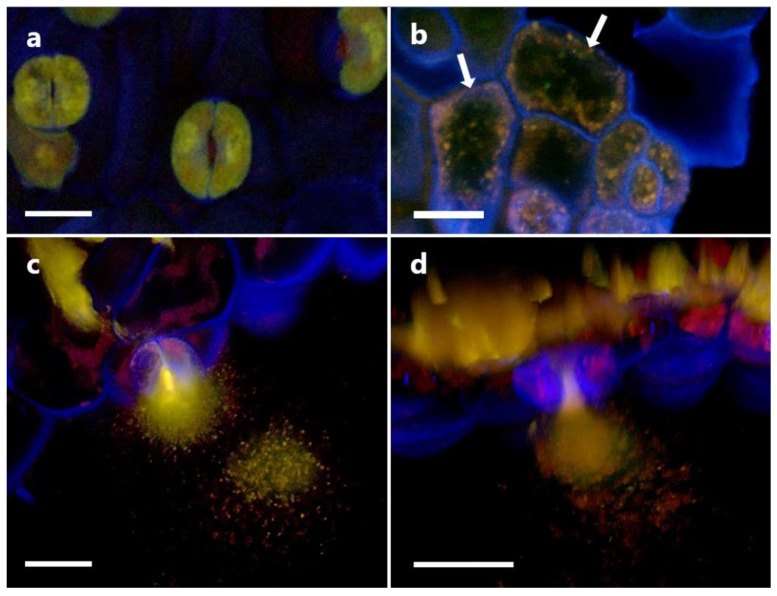
P3AW:*mCherry* being ejected through an open stoma in the cotyledons of inoculated clover plants. (**a**) A 2D image of the stomata and guard cells of an uninoculated clover plant. (**b**) A 2D image of the guard cells of an P3AW:*mCherry*-inoculated clover plant. (**c**) A 2D and (**d**) 3D image of a plume of P3AW:*mCherry* cells escaping from the stoma opening. Scale bar = 20 μm.

We also attempted to determine the localization of P3AW:*mCherry* in the shoot, using the cotyledons. P3AW:*mCherry* was observed in the peripheries of the guard cells, similar to their localization in the roots (Figure 16b). P3AW:*mCherry* was also observed escaping from open stomata (Figure 16c,d), indicating that P3AW:*mCherry* can be found in the shoots.

## 4. Discussion

As the agricultural industry becomes increasingly interested in harnessing the power of biostimulants in lieu of traditional agrochemicals, beneficial microbes originating from plants, such as rhizobacteria, mycorrhizae, and endophytes, have garnered greater attention from the research community. Many plants have been prospected for their beneficial microbes, though desert plants have often been left out of such studies.

In this study, we investigated the endophytic *Pseudomonas* sp. of *A. palmeri*, an agave species native to the Sonoran Desert of the American southwest, and assessed their potential as biostimulants as well as the possible mechanisms behind any biostimulant activity.

Previous findings show that *Pseudomonas* spp. are potent root-growth promoters [50,51], though they can also improve shoot growth [52]. The root- and shoot-length results from testing individual and a synthetic community of *Pseudomonas* sp. strains on Kentucky bluegrass, clover, carrot, and coriander corroborate these results (Figure 3, Figure 5, Figure 7 and Figure 9). Additional evidence of root-growth promotion can also be seen with the increases in soil adsorption around the roots of inoculated seedlings (Figure 2, Figure 4, Figure 6 and Figure 8).

Though no direct data was taken on soil aggregation, the increased amount of soil adsorption suggests that the *Pseudomonas* sp. Is capable of increasing soil aggregation around host roots. Polysaccharides and other metabolites produced by bacteria in the rhizosphere contribute positively to soil aggregation around plant roots [53,54,55,56], so increased soil aggregation may be a byproduct of bacteria metabolism in the rhizosphere. However, an increase in soil aggregation may also be due to increased root exudate production by the host. Previous studies have shown that increased root exudate production is correlated with increased soil aggregation around the root [57,58]. This increase in root exudate production may be caused by exudate leakage as a result of root damage, particularly when *Pseudomonas* sp. are escaping the root hair tips (Figure 14), but it may also be a result of deliberate host selection for beneficial microbes. Plants are known to use root exudates as a way to attract beneficial microbes [59,60,61] and a study on flax (*Linum usitatissinum* L.) and tomatoes has shown that host plants will select for specific soil Pseudomonads instead of the entire *Pseudomonas* soil community [62]. It is possible that the tested host plants are producing more exudates to continuously attract and maintain these beneficial *Pseudomonas* sp. strains in the rhizosphere. 

One possible reason that non-native hosts may be selecting for *Pseudomonas* sp. strains from agave is the production of siderophores. *Pseudomonas* spp. are known to produce iron-chelating compounds known as siderophores, one of which is the fluorescent siderophore pyoverdine [63,64]. Pyoverdine is a yellow-green compound which glows blue under UV light. Four out of the five strains in this study were yellow-green in color and fluoresce blue under UV light (Table 2), which may be a sign that they are producing pyoverdine. Siderophores are known to increase plant growth [65,66,67], which may contribute to preferential host selection for these particular strains. Lemanceau et al.’s research on flax and tomatoes revealed that hosts selected for fluorescent Pseudomonads over other non-fluorescent Pseudomonads [62], lending credence to the idea that non-native hosts are producing root exudates to attract *Pseudomonas* sp. strains.

Other reasons for the selection of agave *Pseudomonas* sp. strains may include their nitrogen-fixing ability or their phosphate solubilization ability. Both nitrogen and phosphorous are essential to plant growth and are abundant in the environment, but they are often in forms that are inaccessible to the plant as atmospheric nitrogen and insoluble phosphorus [68,69]. Nitrogen fixation and phosphate solubilization are methods by which microbes can increase the amount of plant-available nitrogen and phosphorus in the soil and rhizosphere, thus selection for microbes that are able to participate in these processes would be beneficial for the plant. However, strain WC is notable for not being able to produce pyoverdine, fix nitrogen, or solubilize phosphate. This points to another reason that non-native hosts are selecting for agave *Pseudomonas* sp. in place of or in addition to the characteristics listed above. Further investigation into the plant-microbe interactions between agave *Pseudomonas* sp. would help to shed light on this matter.

Nevertheless, depending on the environment they are grown in, plants are able to change the types of microbes they select for. Research into the microbiomes of plants growing in desert regions shows that plants in dry areas are able to naturally acquire drought-resistant microbiomes [70,71]. Since the *Pseudomonas* sp. strains in this study were originally isolated from wild agave, they may have been selected by their native agave host for their ability to increase drought tolerance. However, the tests on wheat subjected to drought stress do not provide any inconclusive results about the ability of *Pseudomonas* sp. to confer greater drought tolerance. Data on root lengths suggests that inoculation with *Pseudomonas* sp. increases root length, and subsequently, water intake, in arid environments (Figure 10), while data on the germination rate suggests that inoculation with *Pseudomonas* sp. harms the ability for seeds to germinate in drought conditions. Perhaps these particular strains are not entirely compatible with the wheat cultivar used. A study on rhizospheric *Pseudomonas aeruginosa* showed that, despite the strain’s plant growth-promoting ability, it inhibited seed germination in wheat and corn [72]. Additionally, a study on *Pseudomonas chlororaphis* revealed that inoculation with *P. chlororaphis* inhibited seed germination in barley and wheat, but not cucumber or rice, and that the inhibition of seed germination was correlated with the strain’s ability to induce systemic disease resistance [73]. We see the same pattern of notable seed germination inhibition in wheat but not in other hosts that were inoculated with *Pseudomonas* sp. from agave, suggesting that the decrease in seed germination is host-species-specific and that decreased seed germination comes at the cost of improving the host’s response to pathogens. In this case, though seed germination may be inhibited, *Pseudomonas* sp. may still prove to be a beneficial microbe by decreasing yield loss from disease.

It is also entirely possible that the results do not match the way in which plants would respond in real-life drought conditions due to the way in which the seedlings were grown in limited volumes of soil inside a drainage-free container. Other studies on the effect of *Pseudomonas* spp. on plant response to salt stress and drought stress conclude that the application of *Pseudomonas* spp. improves growth response in high salt or low water conditions [72,74,75,76,77] and other studies on the application of desert plant microbes to non-desert plants show that inoculation with desert plant microbes will improve the host’s response to drought stress [78,79]. More research, ideally in the field, needs to be conducted to gain further insight into how *Pseudomonas* sp. from agave affects plants’ response to drought and salt stress.

Additional testing to explore the effects of *Pseudomonas* sp. on crops should be conducted over longer periods of time and in the field to determine the true potential and efficacy of the endophytic *Pseudomonas* sp. strains as biostimulants. 

While the application of *Pseudomonas* sp. resulted in growth promotion in the various crop hosts, one point of uncertainty was whether or not the *Pseudomonas* sp. strains were providing the biostimulant effects by entering the host tissues or simply by interacting with the host at the surface level without becoming an endophyte. The inability to track the target microbe once it has been inoculated onto the host plant is one of the main problems facing endophyte research, but one possible solution is to track the microbe is by introducing fluorescent proteins or other markers into the strain of interest. The studies performed with P3AW:*mCherry* show that bacteria transformed with a plasmid to express mCherry are viewable and trackable inside plant tissues using confocal microscopy (Figure 11), making it easy to tell whether or not the strain of interest becomes endophytic, as well as determine where it localizes in host tissues. Other fluorescent proteins, such as green fluorescent protein (GFP) or yellow fluorescent protein (YFP), can potentially be substituted for mCherry if alternative DNA and cell wall stains are used. However, a downside of the plasmid transformation approach is that the host plant must be grown in a substrate containing antibiotics, which limits the tracking studies to the seedling stage due to chloroplast sensitivity to antibiotics. A better approach would be to insert the fluorescent protein production gene directly into the strain’s genome, but this approach would only be feasible if the strain of interest’s genome has already been sequenced. Researchers who wish to conduct preliminary testing on a large variety of newly isolated strains would be better off using plasmid transformation. 

Using the plasmid-transformation approach, we were able to show endophytism and the localization of *Pseudomonas* sp. in tissues and cells (Figure 12, Figure 13, Figure 14, Figure 15 and Figure 16). Previous studies conducted on the rhizophagy cycle via light microscopy have suggested that bacterial endophytes are found in plant cells between the cell wall and the cell membrane [41,42,80]. The results of this study confirm that bacterial endophytes do enter plant cells and can be found near the cell wall (Figure 12 and Figure 16b), though we have not been able to confirm whether or not they are in the space between the cell wall and cell membrane. Further studies need to be conducted with transformed plants displaying fluorescent-protein-tagged membranes to determine if *Pseudomonas* sp. P3AW:*mCherry* or other endophytes are found inside the cytoplasm, between the cell wall and cell membrane, or in both locations. 

Using plasmid transformation and confocal microscopy tracking, we were also able to show that *Pseudomonas* sp. participates in some parts of the rhizophagy cycle, most notably the internalization of bacteria in the roots (Figure 2) and the expulsion of bacteria via the root hairs (Figure 13 and Figure 14). The participation of *Pseudomonas* sp. in the rhizophagy cycle would partially explain its biostimulant properties. *Pseudomonas* spp. are commonly found in the rhizosphere and within plant tissues as endophytes [81,82,83,84,85,86]. As the rhizophagy cycle involves the expulsion of bacterial endophytes into the soil, the ability for this genus to survive and proliferate in soil conditions may make it more valuable as a biostimulant endophyte.

We posit that endophytes which are able to survive outside host tissues may play a greater role in nutrient acquisition and biostimulation due to their ability to participate in the rhizophagy cycle. NaOCl washes are effective at removing many endophytes from plant seeds but fail to remove tightly associated endophytes that cannot be cultured (unpublished data). However, even though these endophytes are still present in the tissues, these disinfected plants do not grow very well, especially when compared to plants that have been re-inoculated with a culturable endophyte (Figure 2 and Figure 4) [30,87]. Even within groups of culturable endophytes, there are certain strains that are more effective at biostimulation than others. Common endophytic biostimulant bacteria include members of the genus *Bacillus* and *Pseudomonas* [88,89,90,91,92], which are both commonly found in soil. It could be that endophytes from genera that are commonly found in soils are more effective as biostimulants compared to endophytes from other genera due to their ability to survive in both root tissues and the rhizosphere soil surrounding it.

Endophyte participation in the rhizophagy cycle may also contribute to nutrient uptake in roots by increasing root hair length, and thus increasing root surface area. The results of this study provide more evidence towards the idea that root endophytes will gather at the tips of root hairs before exiting the root hair tip (Figure 13 and Figure 14), a process that lengthens the root hairs [41,93]. Previous studies implicate reactive oxygen species (ROS) as key signalers during root growth and development, specifically in the formation of root hairs and lateral roots [94,95,96]. While the results of these studies suggest that ROS are the main drivers of root development and root hair formation, it may be that the interaction between ROS and endophytic microbes via the rhizophagy cycle plays an equally large, if not larger, role in root development and nutrient acquisition. For instance, a previous study on nutrient deprivation in *Arabidopsis thaliana* showed that N and K deficiency caused ROS concentrations to increase in the root hairs [97]. This increase in ROS in the root hairs may be the plant’s attempt to degrade more endophytic bacteria to gather additional nutrients as part of the rhizophagy cycle, which then contributes to root hair growth.

Whilst confirming the localization of *Pseudomonas* sp. in plant roots, we found additional evidence to support the idea that *Pseudomonas* sp. were being degraded in the roots. As plants normally move nutrients absorbed through the roots to the shoots using the xylem and phloem, nutrients extracted by bacteria degraded via the rhizophagy cycle cell contents are expected to be visible in the vascular bundle. Indeed, P3AW:*mCherry* cell contents could be seen in the xylem of both the roots (Figure 15b) and shoots (Figure 15c) of inoculated clover, which suggests the nutrients and other bacterial metabolites generated through ROS degradation are transported up into the shoots via the xylem. This adds further credence to the idea that microbes are increasing nutrient uptake in plants not only via secretion-based signaling, but also by releasing their accumulated nutrients via plant-mediated ROS degradation.

**Figure A4:**
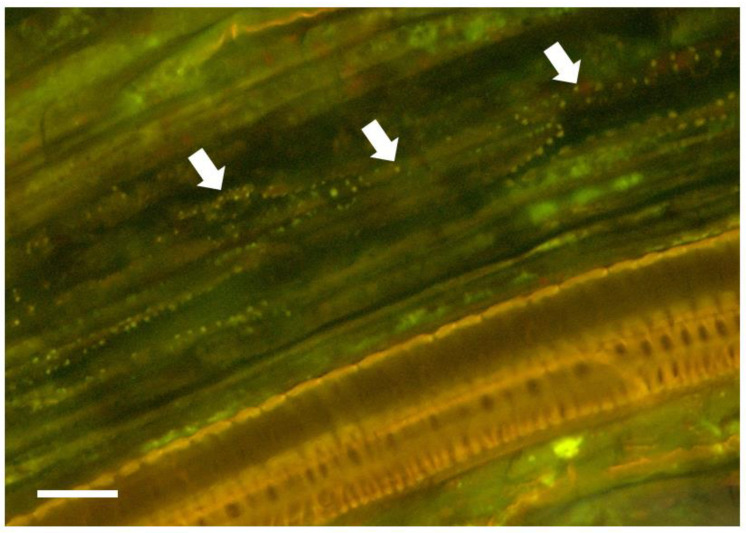
P3AW:*mCherry* in the vascular bundle in inoculated clover roots. Scale bar = 15 μm.

In addition, we also noted the presence of non-degraded P3AW:*mCherry* in the vascular bundle (Figure A4), which may point to bacterial endophytes being exchanged between roots and shoots via the vascular tissues, but may also point to bacterial endophytes using vascular tissues, particularly the phloem, as sugar-rich sanctuaries to reproduce away from ROS degradation. Bacteria may also move via the xylem as well, though we have not been able to see distinct L-forms that indicate intact bacteria in the xylem. More work needs to be performed to determine what kind of a role the vascular tissues play in regards to bacterial endophyte movement and organization within the plant, but the results of this study point to a possible system of endophyte and endophyte-product exchange happening via the vascular tissue of plants, where both endophytes and their cell contents can be moved throughout the plant.

The movement of bacterial endophytes through the plant, particularly via the vascular tissue, may also offer new insights into how endophytes spread amongst plant populations. Currently, it is believed that endophytes are passed vertically from parent to child via the seed, or horizontally from unrelated plants via the soil. However, we were able to catch an instance of endophytes exiting the plant via the stomata, suggesting an aerial route of spread (Figure 16c,d). Similar to how fungal endophytes can use leaves for aerial dispersal, bacterial endophytes may also use leaves for aerial dispersal. Bacteria may exit from open stomata and get carried into neighboring patches of soil, where they can then reproduce and enter new hosts.

## 5. Conclusions

Endophytic *Pseudomonas* sp. isolated from *A. palmeri* improved root length and root soil adsorption when inoculated onto various novel crop hosts. Depending on the host, the strains could also improve shoot length, and there is some evidence that inoculation improves seedling response to drought stress, particularly in the roots. Transformation of the *Pseudomonas* sp. strain P3AW to express the fluorescent protein mCherry verified that it becomes endophytic in the roots and shoots of non-native hosts and confirmed its localization to the cell peripheries, near the cell wall. *Pseudomonas* sp. were photographed congregating near the tips of developing root hairs and in the process of being ejected from the root hair tip, supporting the idea that *Pseudomonas* sp. strains participate in the rhizophagy cycle. Additional photos of free-floating mCherry and DNA in the root and shoot xylem suggest that endophytic *Pseudomonas* sp. or their cell contents are being transported from the roots into the shoots, lending further credence to the hypothesis that the bacterial cells are being degraded for nutrients. The results of this study provide additional evidence of the rhizophagy cycle in plants and how it relates to growth promotion in plants by biostimulant bacteria.

## Figures and Tables

**Table 1:** Primers used in the identification of *A. palmeri* seed endophytes.

Gene	Forward Primer (5′–3′)	Reverse Primer (5′-3′)	Source
16S rRNA	AGAGTTTGATCCTGGCTCAG	CTACGGCTACCTTGTTACGA	White Lab
atpD	CTGGGCCGSATCATGGACG	GTCCATGCCCAGGATSGCG	Hilario, Buckley, and Young [43]
carA	TTCAACACCGCCATGACCGG	TGATGRCCSAGGCAGATRCC	Hilario, Buckley, and Young [43]
recA	TCSGGYAARACCACSCTGAC	RTACCAGGCRCCGGACTTCT	Hilario, Buckley, and Young [43]

**Table 2:** Strains of endophytic bacteria isolated from *A. palmeri*, their characteristics, and GenBank accession numbers.

Strain	Fluorescence	Lipopeptide Production	Casein Digestion	Gelatinase Activity	N Fixation	P Solubilization	16S rRNA GenBank Accession No.
P3AW	✓ (blue-green)	✓	✓	✓	✓	✓	OP584654
P3BW	✓ (blue-green)	✓	✓	✓	✓	✓	OP584655
AY2	✓(blue-green)	✓	✓	✓	✓	✓	OP584656
WC	X	X	X	X	X	X	OP584657
WCY	✓(blue-green)	✓	X	X	X	✓	OP584658

✓ = strain displays the characteristic; X = strain does not display the characteristic.

## Data Availability

All data generated are in the article.

## Supplementary Materials

The following supporting information can be downloaded at: https://www.mdpi.com/article/10.3390/biology11121790/s1. Video S1: Video showcasing a 3D confocal stack of P3AW:*mCherry*-inoculated clover root cap cells, viewed from the side. P3AW:*mCherry* cells (orange globules) can be seen at the peripheries of the cells, near the cell wall. The stack was generated using the Zen 3.6 (blue edition) software (Carl Zeiss). Video S2: Video showcasing a 3D confocal stack of P3AW:*mCherry*-inoculated clover root cap cells, viewed from the front. P3AW:*mCherry* cells (orange globules) can be seen mainly near the peripheries of the cells, near the cell wall. The stack was generated using the Zen 3.6 (blue edition) software (Carl Zeiss).

## Appendix A

**Table A1:** Average number of seeds germinated per magenta box by plant host and by inoculated strain. Each box contained a total of 10 seeds. ± refers to standard deviation.

Plant	Uninoculated	P3AW	P3BW	AY2	WC	WCY	Mix (P3AW, AY2, WC, WCY)
KY Bluegrass	8.33 ± 1.25	8.67 ± 1.25	8.00 ± 0.82	9.67 ± 0.47	7.67 ± 2.62	8.33 ± 0.47	n/a
Clover	5.00 ± 1.41	6.00 ± 1.63	8.00 ± 1.63	7.33 ± 0.47	6.00 ± 0.81	6.67 ± 0.47	n/a
Carrot	6.67 ± 1.25	n/a	n/a	n/a	n/a	n/a	8.33 ± 1.70
Coriander	7.00 ± 1.41	n/a	n/a	n/a	n/a	n/a	6.33 ± 0.47

**Table A2:** Average number of wheat seeds germinated per magenta box by treatment. Each box contained a total of 10 seeds. ± refers to standard deviation.

Treatment	Irrigated, Uninoculated	Irrigated, Inoculated	Drought, Uninoculated	Drought, Inoculated
Average # of Germinated Seeds Per Box	9.25 ± 0.43	7.75 ± 1.79	8.00 ± 1.87	5.50 ± 1.12
Average # of Dead Seedlings Per Box	0	0	3.25 ± 1.48	3.25 ± 0.43
